# Temporal associations between depressive symptoms, self-esteem, and satisfaction with family life: A 15-year study

**DOI:** 10.3389/fpubh.2023.1144776

**Published:** 2023-03-13

**Authors:** Mohsen Joshanloo

**Affiliations:** Department of Psychology, Keimyung University, Daegu, Republic of Korea

**Keywords:** depressive symptoms, self-esteem, family life satisfaction, within-person, KOWEPS

## Abstract

**Introduction:**

In South Korea, depression has significant economic and social impacts, including increased healthcare costs and a relatively high suicide rate. Reducing the prevalence of depressive symptoms in the general population is therefore an important public health goal in this country. To achieve this goal, it is essential to identify the factors that may increase or decrease the risk of depression. This study examined the association between depressive symptoms and two indicators of wellbeing: self-esteem and satisfaction with family life. A primary objective was to examine whether higher self-esteem and satisfaction with family life could predict a decrease in depressive symptoms in the future.

**Methods:**

A large representative sample was used, collected over a 15-year period with annual lags. The random intercept cross-lagged panel model was used to examine reciprocal associations between the 3 variables at the within-person level.

**Results:**

All within-person effects were found to be reciprocal, significant, and in the expected direction. Thus, within-person deviations in any of the variables are associated with future within-person deviations in the other variables.

**Discussion:**

These results suggest that indicators of positive mental health (self-esteem and satisfaction with family life) are protective factors against future depressive symptoms. In addition, depressive symptoms are risk factors for lower self-esteem and lower satisfaction with family life.

## Introduction

The prevalence of depression is increasing worldwide (1). The COVID-19 pandemic has also exacerbated the already high burden of depression (2). In Korea in particular, the prevalence of depression is increasing, depression costs billions of dollars in lost GDP, and contributes to the country's high suicide rate (3–5). Therefore, reducing depressive symptoms in the general population is an important public health goal. Factors that protect against depressive symptoms need to be identified and emphasized in strategies and interventions. For example, self-esteem and solid family ties are considered protective factors against depression (6). On the other hand, depression itself contributes to a whole range of mental health problems, including a reduction in the level of its protective factors. Therefore, the interplay between depressive symptoms and these protective factors needs to be closely examined in order to make informed policy decisions and develop interventions. The present study examined the reciprocal relationships between depressive symptoms, self-esteem, and satisfaction with family life in a large national sample from the Korean Welfare Panel Study (KOWEPS) collected over a 15-year period. The following is an overview of prior research on the relationships between these variables.

### Relationship satisfaction and depressive symptoms

While relationship dissatisfaction and a lack of social support are recognized as risk factors for depressive symptoms, good relationships are considered a buffer against depressive symptoms (7, 8). Not only do current relationships affect a person's mood, but the quality of relationships in early childhood also predicts the future occurrence of mood disorders (9, 10). High-quality relationships mitigate the negative effects of emotional distress, acting as a buffer against mood dysregulation (11).

Significant cross-sectional correlations between indicators of relationship satisfaction and depression are usually interpreted as demonstrating the predictive power of relationship quality in determining the occurrence of depression. However, depressive symptoms may also influence future relationship quality, as shown in some studies. In a longitudinal study, Morgan et al. (12) found that depressive symptoms contributed to a future decline in relationship satisfaction. In another longitudinal study, brooding was found to prospectively predict decreased relationship satisfaction (13). Another study found that social connection did not significantly predict emotional wellbeing (including negative affect) in the future. Rather, it was emotional wellbeing that predicted future levels of social connection (14). Some longitudinal studies have documented a reciprocal relationship between relationship satisfaction and depressive symptoms (15, 16). Thus, a mutual relationship between depressive symptoms and relationship satisfaction is plausible.

### Self-esteem and depressive symptoms

Self-esteem and depression are closely related. In fact, low self-esteem is a diagnostic criterion for depressive disorders (17). According to cognitive vulnerability models of depression (18), personal perceptions of unworthiness pave the way for depressive symptoms. Research also suggests that high self-esteem is a buffer against depressive symptoms (19). The scar model, on the other hand, predicts that depression leads to a decline in self-esteem because depression affects personal resources and leaves scars on the person's self-concept that gradually undermine self-worth (20). Some longitudinal studies of the vulnerability and scar models have found more support for the vulnerability model (20–22), suggesting that self-esteem is a robust predictor of future depressive symptoms. However, these studies did not partition the variance into within-person and between-person components and did not estimate purely within-person estimates. Conceptually, the relationship can run in either direction, which is why this study examined the mutual within-person associations between the two variables.

### Self-esteem and relationship satisfaction

Self-esteem and relationship satisfaction are also linked (23, 24). Sociometer theory (25) posits that self-esteem is a means by which people measure how important they think they are to others and how much they believe they are socially accepted. From this perspective, relationship satisfaction would be the predictor and self-esteem would be the outcome. Leary (25) provides a compelling review of the empirical evidence supporting this prediction. However, self-esteem itself may also be a predictor of relationship satisfaction. According to the dependence regulation model (26), a person's self-esteem plays a critical role in satisfaction in romantic relationships. Individuals with low self-esteem tend to respond to relationship threats with self-destructive and relationship-destructive activities that can lead to the failure of their relationships (27). A lack of perceived self-esteem leads to feelings of not being accepted and loved by one's romantic partner, which can lead to distancing from and devaluing the partner (28). A meta-analysis of longitudinal studies has shown that self-esteem and relationship satisfaction are reciprocally related (29). Accordingly, this study examined the mutual relationship between the two variables.

### Self-esteem, relationship satisfaction, and depressive symptoms

All three variables are cross-sectionally related and there is evidence of longitudinal relationships between them. However, not many previous studies have looked at all three variables together, with a clear lack of longitudinal studies addressing this issue. One cross-sectional study showed that self-esteem was more strongly correlated with depression than with relationship satisfaction (30). A longitudinal study also showed that self-esteem was a better predictor of depression than relationship outcomes (31). Thus, a conclusion from previous research might be that self-esteem and depression are more strongly related to each other than to relationship satisfaction. However, this conclusion is far from decisive, and further longitudinal, particularly within-person, studies are needed to reach more solid conclusions.

### Limitations of the previous studies

The previous studies have provided invaluable insights into the relationships among the three variables. However, they have some limitations, some of which are highlighted here. Firstly, to date, almost all studies have focused primarily on the correlations between two of the variables, with only a few examining the relationships among all three variables simultaneously. It has proven difficult to integrate information about the relationships between variables that come from separate studies. For example, two separate studies may show that both relationship satisfaction and self-esteem are significant prospective predictors of depressive symptoms. However, the findings across the two studies may say little about the relative importance of the two predictors. Moreover, it is not clear whether all variables remain significant predictors of each other when they are simultaneously included as regressors in a single model.

Secondly, many of the published studies on this topic are cross-sectional. Therefore, they say little about temporal dynamics. Many cross-sectional studies assume that one variable (e.g., self-esteem) predicts the other variables (e.g., depressive symptoms) without considering the possibility of a reciprocal relationship between the two variables. Cross-sectional studies do not provide reliable information about the temporal order (i.e., precedence and directionality) between the variables either. Therefore, we need more longitudinal studies to shed light on the direction of the associations between these three variables. Finally, longitudinal research in this area has not generally distinguished between within-person (intra-individual) and between-person (inter-individual) sources of variation. When the two levels are not separated, it is unclear how much of the estimated effects reflect synchronicity and how much reflects directional within-person associations. This drawback casts doubt on our assumptions about the directionality of the effects observed in previous studies. Few studies have examined the associations between these variables at the within-person level. For example, one study that disaggregated within-person and between-person variance found support for the vulnerability model (32), while Braun et al. (33) found no within-person lagged associations between depression and self-esteem. Thus, a comprehensive within-person understanding of the relationships among these variables has yet to be established.

### The present study

This study sought to address all of these limitations using a large Korean data set collected over 15 years with annual lags. Rather than assuming the direction of the relationships, all possible reciprocal effects were examined in this study. Investigating the three variables simultaneously allows us to determine the relative importance of the predictors for each of the outcomes. For example, it would be possible to determine which of the two factors, self-esteem and relationship satisfaction, is a stronger predictor of future levels of depressive symptoms. The study used a statistical procedure that partitions the variance into within-person and between-person sources, as explained in more detail below. This allows for the estimation of lagged within-person effects that unravel the direction of associations between variables.

As described in the methods section, the self-esteem and depression measures used in KOWEPS are widely used across the globe. The KOWEPS also includes an item measuring overall satisfaction with family life, which was used in this study. Relationalism is considered a central theme underlying Korean collectivism. This characteristic is commonly attributed to the influence of Confucianism, as noted in a review by Park and Han (34). In Korea, strong family ties are considered a key component of interpersonal functioning and serve as a reliable indicator of overall relationship satisfaction (35).

### Analytical approach

Much of the psychological literature is based on cross-sectional data, focusing on between-person associations. For example, a negative cross-sectional association between self-esteem and depressive symptoms suggests that individuals with low self-esteem are more likely to suffer from depressive symptoms. However, this type of analysis does not reveal the temporal sequence of the variables and their longitudinal interplays. Another level at which the relationships among these variables can be examined is the within-person level, which requires longitudinal data and accounts for intraindividual changes over time (36). At the within-person level, the question would be whether a change in one variable is related to a future change in the same variable or another variable. Technically, knowing the correlations between two variables at the between-person level does not tell us anything about their associations at the within-person level (37), and a lack of equivalence between the two levels can occur due to a variety of factors (38). The main purpose of this study was to use longitudinal data to analyze the direction of associations between the three variables in the study. Since the directionality of associations between variables can only be studied at the within-person level, the focus of this study is on within-person effects (39). Specifically, the study sought to determine if a within-person increase or decrease in one variable is related to a within-person increase or decrease in the other variables in the future.

The Cross-Lagged Panel Model (CLPM) is a common technique for analyzing longitudinal data (40). The main purpose of the CLPM is to assess the relationships between two variables over time, taking into account the past values of each variable (39). The CLPM has been criticized for assuming that individuals change over time only relative to the group average, ignoring the fact that people change longitudinally around their personal averages as well (41). Because CLPM cannot distinguish between the sources of variance between and within individuals, its results can be difficult to understand and interpret. This is especially true when the focus of a study is on within-person relationships (40, 42). To overcome these drawbacks, this study used the Random Intercept Cross-Lagged Panel Model (RI-CLPM). This model is an extension of the basic CLPM, which distinguishes between stable differences between individuals around the grand mean and within-person deviations from the personal means (43). The within-person component of the RI-CLPM can examine whether a within-person deviation from the typical or expected level of a variable is associated with deviations in the same variable or other variables at the next time point. Thus, the RI-CLPM can clarify the direction of associations between two variables.

## Methods

### Sample

Data are from the Korea Welfare Panel Study (KOWEPS), conducted by the Korea Institute for Health and Social Affairs and the Seoul National University Institute of Social Welfare (the current author is not affiliated with either of the institutions). The target population of KOWEPS is all households living across the republic of South Korea (however, Islands and special facilities are excluded). The KOWEPS is an annual longitudinal panel survey that began in 2006. However, the 2006 wave was excluded from the present study, because the family satisfaction question was not asked in that wave. This study uses data from waves 2–16 (2007–2021), hereafter called time points 1–15. Information about the survey and access to the data and materials can be found on the study's website (https://www.koweps.re.kr:442/main.do). Out of the 27,582 participants in the KOWEPS aggregated dataset (waves 1–16), 20,973 have responded at least once to the variables of this study during waves 2–16 and were included in the analysis. The average age was 48.622 in 2007 (Median = 47.000, SD = 21.089, females = 53.8%).

### Measures

#### Depressive symptoms

An 11-item form of the Center for Epidemiological Studies Depression (CES-D) symptoms index was used to measure depressive symptoms (44). The scale asks participants to report how often they experienced 11 symptoms of depression over the past week, on a 4-point scale from 1 = *Rarely or none of the time (*<*1 day)* to 4 = *Mostly (5–7 days or more a week)*. A principal axis factoring analysis of the 11 items in the first time point confirmed that a one-factor model is consistent with the data. The first three initial eigenvalues were 4.910, 1.361, and 0.832. The amount of variance explained by a single factor was 39.582%. The factor loadings ranged between 0.406 and 0.779. The items were averaged to construct a composite depression score. Considering that the kurtosis values for the original depression scores were >2 for some of the waves, the depression scores were log-transformed in this study to reduce the degree of non-normality.

#### Self-esteem

The Korean version of the Rosenberg Self-Esteem Scale (45) was used. The 10 items are rated on a 4-point scale from 1 = *never* to 4 = *always*. An initial analysis revealed that one of the items (“I wish I could have more respect for myself”) is not positively correlated with the rest of the items (item-total scale *r* = −0.206). Factor analysis also showed that the items had a negative loading on the latent variable of self-esteem. Therefore, this item was left out of this study. A principal axis factoring analysis of the remaining nine items in the first time point confirmed that a one-factor model is consistent with the data. The first three initial eigenvalues were 3.500, 1.091, and 0.910, and the amount of variance explained by a single factor was 31.592%. Factor loadings ranged between 0.418 and 0.696.

#### Family life satisfaction

The respondents were asked to indicate how satisfied they were with their family life in the current year. The item was rated on a scale from 1 = *very dissatisfied* to 7 = *very satisfied*.

### Attrition and missing data handling procedures

Of the 20,973 participants who responded to at least one variable and were included in the study, 5,236 (25%) participated in all 15 waves used in this study, and 15,737 participants (75%) had at least one missing wave. The maximum likelihood estimation used in this study provides an efficient model-based strategy to use all available data. Maximum likelihood under missing data theory does not discard participants with incomplete data. Instead, it utilizes all available data to identify the parameter values (46). A longitudinal missing data indicator was calculated, showing the number of missing waves for each participant (ranging between 0 and 14). As shown in **Table 2**, this variable had very weak correlations with the variables of the study at the first time point (the correlations with variables at the other time points were also comparable, and not reported due to space restraints). Overall, this suggests that individuals with worse mental health (higher depressive symptoms and lower self-esteem and family life satisfaction) were slightly more likely to have a lower participation frequency. As a supplementary missing data management strategy, this missing data indicator was added as an auxiliary variable to the model. Auxiliary variables carry information about missingness or variables with missing values but are not part of the main model. They are used to fine-tune an analysis with incomplete data by improving precision in parameter estimation (46).

### Statistical analysis

A random-intercept cross-lagged panel model with robust maximum likelihood (MLR) was performed in Mplus. All three focal variables were included in this model. The observed variables were regressed on the control variables of age (calculated for 2021) and gender at all time points. The longitudinal missing data indicator was included in the model as an auxiliary variable. The autoregressive and cross-regressive paths were restricted to equality across time points. A Comparative Fit Index (CFI) value of >0.95, an Root Mean Square Error of Approximation (RMSEA) value of < 0.07, and a Standardized Root Mean Square Residual (SRMR) value of < 0.08 were used as thresholds for a good model fit in this study (47).

## Results

### Descriptive statistics

The descriptive statistics for the variables of the study and Cronbach's alphas (at the first time point) are reported in Table 1. Descriptive statistics for the variables at other time points are reported in the Supplementary material. The degree of non-normality was considered small based on the proposed thresholds of skewness > 2 and kurtosis > 7 for severe non-normality in structural equation modeling (48). The robust maximum likelihood estimator used in this analysis is a suitable approach for analyzing the data because it is quite robust to small deviations from normality. Table 2 shows the intercorrelations at the first time point, and it can be seen that none of the correlations exceed 0.70, indicating that collinearity is not a problem in the data (49).

**Table 1:** Cronbach's alphas and descriptive statistics.

	**Alpha**	**Min**	**Max**	**M**	**SD**	**Skewness**	**Kurtosis**
Depressive symptoms	0.870	0.00	1.39	0.374	0.3149	0.627	−0.479
Self-esteem	0.800	1.00	4.00	3.034	0.5195	−0.500	0.018
Family life satisfaction	–	1.00	7.00	5.256	1.348	−0.774	−0.050

**Table 2:** Correlation matrix (time point 1).

	**1**	**2**	**3**
1. Depressive symptoms	1		
2. Self-esteem	−0.558^***^	1	
3. Family life satisfaction	−0.399^***^	0.434^***^	1
4. Number of missing waves	0.036^***^	−0.061^***^	−0.024^*^

^***^p < 0.001, ^*^p < 0.05.

### Random intercept cross-lagged panel model

The RI-CLPM with all the variables fitted the data very well, Chi-Square = 4,269.227, degree of freedom = 930, *p* < 0.001, RMSEA = 0.013, 90% confidence interval for RMSEA = 0.013–0.013, CFI = 0.985, SRMR = 0.024. The relevant parameter estimates are reported in Table 3. All the auto-regressive effects are significant. This means that the within-person deviations persist to the next time point. For example, a higher-than-expected score of self-esteem at one time point is associated with a higher-than-expected score of self-esteem at the next time point.

**Table 3:** Parameter estimates for RI-CLPM.

**Predictor**	**Outcome**	**Unstandardized coefficient**	***p***	**95% CI**		**Standardized coefficient**
				**Low**	**Up**	
**Auto-regressive**						
Depressive	Depressive	0.151	0.000	0.143	0.158	0.163
Self-esteem	Self-esteem	0.142	0.000	0.135	0.149	0.155
Family satisfaction	Family satisfaction	0.119	0.000	0.112	0.126	0.126
**Cross-lagged**						
Self-esteem	Depressive	−0.017	0.000	−0.021	−0.013	−0.029
Family satisfaction	Depressive	−0.003	0.000	−0.005	−0.002	−0.016
Family satisfaction	Self-esteem	0.009	0.000	0.007	0.011	0.027
Depressive	Self-esteem	−0.067	0.000	−0.078	−0.057	−0.046
Depressive	Family satisfaction	−0.105	0.000	−0.134	−0.077	−0.024
Self-esteem	Family satisfaction	0.067	0.000	0.050	0.084	0.025
**Trait covariances**						
Depressive	Self-esteem	−0.034	0.000	−0.035	−0.033	−0.835
Self-esteem	Family satisfaction	0.128	0.000	0.123	0.133	0.742
Family satisfaction	Depressive	−0.067	0.000	−0.070	−0.064	−0.701
**State covariances (time point 1)**						
Depressive	Self-esteem	−0.042	0.000	−0.044	−0.039	−0.394
Self-esteem	Family satisfaction	0.144	0.000	0.133	0.156	0.297
Family satisfaction	Depressive	−0.077	0.000	−0.084	−0.070	−0.257
**State covariances (time point 2)**						
Depressive	Self-esteem	−0.027	0.000	−0.029	−0.025	−0.312
Self-esteem	Family satisfaction	0.108	0.000	0.099	0.117	0.266
Family satisfaction	Depressive	−0.052	0.000	−0.058	−0.046	−0.204

CI, confidence interval. All regressive paths are held equal across waves. The standardized regression coefficients are related to the paths between time points 1 and 2. Regression coefficients related to other waves are similar to the reported coefficients and are not reported here due to space limitations. State covariances related to other waves are similar to the reported ones and are not reported here.

The focus of the present study is on the cross-regressive effects. Are within-person deviations in a variable related to within-person deviations in the other variables at the next time point? The results show that all three variables are mutually associated at the within-person level in the expected direction. Depression is negatively associated with self-esteem and family life satisfaction, and the latter two are positively associated. Thus, for example, a higher-than-expected score of depression at one time point is associated with a lower-than-expected score of self-esteem and relationship satisfaction at the next time point. Orth et al. (50) have provided guidelines for interpreting standardized cross-lagged effects in the RI-CLPM. They suggest that, a standardized cross-lagged effect of 0.03 represents a small effect, 0.07 represents a medium effect, and 0.12 represents a large effect. Applying these guidelines to the current results, all cross-lagged effects can be considered small, except the effect from depression to self-esteem, which can be considered small to moderate in size.

Three types of non-temporal associations are reported in Table 3. The between-person associations (correlations between the trait components) were all strong and significant. The covariances between the state components at the first time point were comparable to the covariances between residuals of the state components at the second time point. All of the non-temporal within-person correlations were weak to moderate and significant. The covariances at other times points were similar to those at the second time point and are not reported due to space constraints.

## Discussion

Depressive symptoms affect both emotional and functional components of life, and thus monitoring their prevalence provides a valid assessment of overall wellbeing in the general population (51). This study focused on the temporal dynamics involved in the interplay between depressive symptoms and two of their protective factors: self-esteem and family life satisfaction. All of the within-person effects were found to be mutual, significant, and in the expected direction. These results suggest that indicators of positive mental health (self-esteem and family life satisfaction) are protective factors against future depressive symptoms in the general population. Furthermore, depressive symptoms are risk factors for lower future self-esteem and family life satisfaction.

### Self-esteem and family life satisfaction as predictors

The results showed that both self-esteem and family life satisfaction predicted lower future levels of depressive symptoms. The effect sizes were both small, but slightly larger for self-esteem. Previous research indicates that the relative emphasis placed on self-esteem and family ties varies by culture. According to cultural psychologists, Western cultures place greater importance on self-esteem, and self-esteem is more advantageous in these cultures. In contrast, East Asian cultures place more emphasis on family relationships, and these cultures are thought to benefit more from high-quality family relationships than from high self-esteem. Self-esteem is downplayed in Asian contexts and is not considered a central motivational goal (52–54). Based on these assumptions, it would be predicted that in Korea, self-esteem would be a weaker predictor of depressive symptoms than the quality of family relationships. However, the present results suggest that self-esteem is a slightly better predictor of future depressive symptoms than satisfaction with family life.

The assumption that self-esteem is not an important player in collectivistic cultures is largely based on the results of cross-sectional studies. The present study used a large representative data set and a within-person approach, and hence the results are more likely than cross-sectional studies to reflect the true temporal associations between the variables. This is an illustration of the fact that when large longitudinal data sets and rigorous statistical procedures are used, conventional wisdom can prove to be wrong. It is noteworthy that this study is not unique in challenging the assumption of the irrelevance of self-esteem in Korean culture. For example, in a longitudinal study, In (55) found that the effect of self-esteem on subsequent happiness was stronger than that of happiness on subsequent self-esteem. Other studies also show that family ties are not the most important value for Korean people, as many would assume. For example, in a survey of 17 wealthy countries, Korea was the only country in which material wealth was most frequently mentioned as a source of meaning in life, while family was the most frequently mentioned source in 14 other countries (56).

The findings are consistent with previous research suggesting that positive mental health, as indicated by positive qualities such as self-esteem and relationship satisfaction, is protective against mental and physical disorders (57, 58). Accordingly, the findings may inform the development of interventions and new policies to prevent depressive symptoms (and likely other mood and anxiety disorders). Interventions focused on self-esteem have been used in other cultures to combat depressive symptoms. For example, Hilbert et al. (59) focused on increasing self-esteem to treat depressive symptoms as part of cognitive behavioral therapy for 147 German psychiatric inpatients. They found that the more self-esteem was increased, the more depressive symptoms decreased. Therefore, investing in interventions that target different aspects of positive mental health (e.g., self-esteem and relationship skills) appears to be an effective preventive measure against depressive symptoms. It should be noted, however, that this study was not an experimental study with a sufficient level of experimental control and therefore causality cannot be assumed. However, the study shows that an increase in positive skills is followed by a decrease in depressive symptoms. The increase in positive skills can be accelerated by effective interventions and supportive measures and resources.

### Depressive symptoms as a predictor

Recently, attention has focused on the consequences of depressive symptoms, rather than viewing depression merely as an outcome (60). On this basis, the present study investigated all possible mutual associations between the variables without imposing any theoretical and/or statistical constraints on directionality. The result showed that depressive symptoms were predicted by and predictive of levels of self-esteem and satisfaction with family life. Notably, the largest effect size in this study was for the within-person path from depressive symptoms to self-esteem. Although both the vulnerability and scar models were supported, the scar model receives more support in this Korean sample. Therefore, depressive symptoms should not only be considered as an outcome, but they also have predictive power for other indicators of wellbeing over time.

These findings have a clear implication: prevention is better than treatment. Protective factors such as self-esteem and relationship skills are negatively affected by experiencing depressive symptoms themselves. This suggests that strengthening these protective factors becomes more difficult after the onset of depressive symptoms. Therefore, the most effective strategy would be to enhance these factors before depressive symptoms occur. To do this, it is necessary to identify vulnerable groups in Korean society and intervene in advance.

### Between- and within-person associations

The between-person associations were strong and in the expected direction. The fact that self-esteem was more strongly associated with depressive symptoms than was satisfaction with family life contradicts assumptions that family relationships take precedence over self-esteem in Asian countries [(61), for a review see (62)], even at the between-person level. Therefore, this finding from a collectivistic country (63) deserves the attention of cultural psychologists. The main focus of this study was on within-person associations. The new focus on within-person processes (42) has led many researchers to rethink commonly accepted notions and to empirically re-examine relationships between variables after variance decomposition [e.g., (64)]. Within-person results may be at odds with cross-sectional results. For example, although many cross-sectional studies have found a positive relationship between religiosity and life satisfaction (65), two recent studies have found non-significant within-person relationships between religiosity and life satisfaction (66, 67). The main difference between the results of the present study and most cross-sectional studies is that this study explicitly examined the directionality of the relationships rather than relying on theoretical expectations. The present study is among the few studies that have examined the relationship between these three variables at the temporal within-person level. Before we can draw definitive conclusions about the within-person relationships between these variables, we need further with-person studies.

## Limitations and concluding remarks

The study had some limitations that must be acknowledged. For example, the scale used in this study for satisfaction with family life contains only one item. Future studies need to use more reliable instruments to measure this concept in Korea. The KOWEPS uses the survey method to collect data, which may limit the depth and breadth of the findings. Although long-term surveys provide valuable data and insights, they have their own limitations, such as the possibility of response bias and reliance on self-reporting. To overcome the limitations of the current study, future research could take a multimethod approach by incorporating multiple data sources such as interviews, informant assessments, experiments, and observational data. This would allow for a more comprehensive understanding of the phenomenon under study, as each data source can contribute unique perspectives and insights. In addition, combining multiple methods can increase the validity and reliability of the results and provide a more solid basis for conclusions.

The strength of associations between variables is to some extent a function of the time lag between measurement time points (68). Therefore, the results of longitudinal studies should be interpreted by considering the lag length used. In this study, the lag length is 1 year. Therefore, the present results reflect the long-term relationships among the studied variables. The present results do not indicate how these variables covary on an hourly or daily basis. Studies with shorter lags (e.g., experience sampling, daily diaries, or other longitudinal studies with a lag of 1 week or 1 month) might find stronger or weaker associations among the variables. Accordingly, the present results are most informative in designing relatively long-term interventions and policies. Finally, the results presented here are based on a general population data set, and thus the results are particularly useful in understanding the interplay of these variables in the general population. The applicability of the results to clinical samples and cases requires additional clinical judgment.

Despite its limitations, this study has important strengths, including the use of a large representative sample and a long time span. In addition, the study used a methodology that separates between- and within-person levels, allowing for a more nuanced understanding of the temporal relationships between depressive and positive symptoms. The results of this study provide new insights into the complex dynamics of mental health and may help in the development of interventions and strategies to effectively prevent depression and related problems, such as suicide, in Korea.

## Data availability statement

Publicly available datasets were analyzed in this study. This data can be found here: https://www.koweps.re.kr:442/main.do.

## Ethics statement

Ethical review and approval was not required for the study on human participants in accordance with the local legislation and institutional requirements. The patients/participants provided their written informed consent to participate in this study.

## Author contributions

The author confirms being the sole contributor of this work and has approved it for publication.
