# Supplementary material for: Temporal associations between depressive symptoms, self-esteem, and satisfaction with family life: A 15-year study
Source: Front Public Health. 2023 Mar 13;11:1144776. doi: 10.3389/fpubh.2023.1144776 (PMC10040576; doi:10.3389/fpubh.2023.1144776)
Supplement: Supplementary file 1 [file Table_1.DOCX]

| **Descriptive Statistics** | | | | | | | | | |
| --- | --- | --- | --- | --- | --- | --- | --- | --- | --- |
|  | N | Minimum | Maximum | Mean | Std. Deviation | Skewness | | Kurtosis | |
|  | Statistic | Statistic | Statistic | Statistic | Statistic | Statistic | Std. Error | Statistic | Std. Error |
| Depressive01 | 12587 | .00 | 1.39 | .374 | .315 | .627 | .022 | -.479 | .044 |
| Depressive02 | 12194 | .00 | 1.36 | .341 | .296 | .690 | .022 | -.310 | .044 |
| Depressive03 | 11973 | .00 | 1.39 | .331 | .293 | .755 | .022 | -.164 | .045 |
| Depressive04 | 11549 | .00 | 1.39 | .292 | .289 | 1.013 | .023 | .438 | .046 |
| Depressive05 | 10914 | .00 | 1.39 | .272 | .277 | 1.020 | .023 | .481 | .047 |
| Depressive06 | 13945 | .00 | 1.29 | .247 | .269 | 1.058 | .021 | .417 | .041 |
| Depressive07 | 13583 | .00 | 1.39 | .252 | .277 | 1.107 | .021 | .537 | .042 |
| Depressive08 | 13259 | .00 | 1.32 | .284 | .280 | .920 | .021 | .098 | .043 |
| Depressive09 | 12838 | .00 | 1.36 | .237 | .276 | 1.188 | .022 | .660 | .043 |
| Depressive10 | 12309 | .00 | 1.39 | .233 | .276 | 1.216 | .022 | .776 | .044 |
| Depressive11 | 12034 | .00 | 1.39 | .229 | .270 | 1.267 | .022 | 1.056 | .045 |
| Depressive12 | 11704 | .00 | 1.36 | .235 | .274 | 1.187 | .023 | .728 | .045 |
| Depressive13 | 11355 | .00 | 1.36 | .229 | .273 | 1.251 | .023 | .862 | .046 |
| Depressive14 | 10758 | .00 | 1.36 | .243 | .269 | 1.182 | .024 | .790 | .047 |
| Depressive15 | 10545 | .00 | 1.39 | .259 | .279 | 1.101 | .024 | .531 | .048 |
| Self-esteem01 | 12585 | 1.00 | 4.00 | 3.034 | .520 | -.500 | .022 | .018 | .044 |
| Self-esteem02 | 12194 | 1.00 | 4.00 | 3.109 | .486 | -.505 | .022 | .117 | .044 |
| Self-esteem03 | 11973 | 1.00 | 4.00 | 3.069 | .490 | -.476 | .022 | .039 | .045 |
| Self-esteem04 | 11549 | 1.11 | 4.00 | 3.116 | .486 | -.689 | .023 | .475 | .046 |
| Self-esteem05 | 10914 | 1.00 | 4.00 | 3.092 | .482 | -.582 | .023 | .288 | .047 |
| Self-esteem06 | 13945 | 1.11 | 4.00 | 3.090 | .489 | -.447 | .021 | .008 | .041 |
| Self-esteem07 | 13583 | 1.00 | 4.00 | 3.106 | .493 | -.539 | .021 | .156 | .042 |
| Self-esteem08 | 13259 | 1.11 | 4.00 | 3.072 | .470 | -.579 | .021 | .135 | .043 |
| Self-esteem09 | 12838 | 1.00 | 4.00 | 3.084 | .448 | -.565 | .022 | .333 | .043 |
| Self-esteem10 | 12309 | 1.11 | 4.00 | 3.115 | .461 | -.563 | .022 | .378 | .044 |
| Self-esteem11 | 12034 | 1.00 | 4.00 | 3.107 | .456 | -.670 | .022 | .543 | .045 |
| Self-esteem12 | 11704 | 1.00 | 4.00 | 3.136 | .449 | -.563 | .023 | .495 | .045 |
| Self-esteem13 | 11355 | 1.00 | 4.00 | 3.161 | .469 | -.584 | .023 | .368 | .046 |
| Self-esteem14 | 10758 | 1.00 | 4.00 | 3.140 | .447 | -.684 | .024 | .506 | .047 |
| Self-esteem15 | 10545 | 1.00 | 4.00 | 3.144 | .471 | -.669 | .024 | .553 | .048 |
| Family satisfaction01 | 11126 | 1.00 | 7.00 | 5.256 | 1.348 | -.774 | .023 | -.050 | .046 |
| Family satisfaction02 | 12111 | 1.00 | 7.00 | 5.354 | 1.299 | -.950 | .022 | .409 | .045 |
| Family satisfaction03 | 11867 | 1.00 | 7.00 | 5.332 | 1.259 | -.878 | .022 | .386 | .045 |
| Family satisfaction04 | 11463 | 1.00 | 7.00 | 5.272 | 1.224 | -1.069 | .023 | .834 | .046 |
| Family satisfaction05 | 10847 | 1.00 | 7.00 | 5.327 | 1.187 | -1.081 | .024 | .910 | .047 |
| Family satisfaction06 | 13802 | 1.00 | 7.00 | 5.351 | 1.160 | -1.167 | .021 | 1.134 | .042 |
| Family satisfaction07 | 13438 | 1.00 | 7.00 | 5.289 | 1.171 | -1.054 | .021 | .925 | .042 |
| Family satisfaction08 | 13105 | 1.00 | 7.00 | 5.224 | 1.156 | -.958 | .021 | .673 | .043 |
| Family satisfaction09 | 12682 | 1.00 | 7.00 | 5.327 | 1.131 | -1.182 | .022 | 1.284 | .043 |
| Family satisfaction10 | 12159 | 1.00 | 7.00 | 5.398 | 1.101 | -1.204 | .022 | 1.453 | .044 |
| Family satisfaction11 | 11883 | 1.00 | 7.00 | 5.356 | 1.107 | -1.176 | .022 | 1.396 | .045 |
| Family satisfaction12 | 11559 | 1.00 | 7.00 | 5.407 | 1.098 | -1.269 | .023 | 1.600 | .046 |
| Family satisfaction13 | 11208 | 1.00 | 7.00 | 5.490 | 1.090 | -1.297 | .023 | 1.946 | .046 |
| Family satisfaction14 | 10603 | 1.00 | 7.00 | 5.493 | 1.076 | -1.258 | .024 | 1.580 | .048 |
| Family satisfaction15 | 10381 | 1.00 | 7.00 | 5.487 | 1.110 | -1.215 | .024 | 1.548 | .048 |
| Valid N (listwiself-esteem) | 4625 |  |  |  |  |  |  |  |  |
